# Global host molecular perturbations upon in situ loss of bacterial endosymbionts in the deep-sea mussel *Bathymodiolus azoricus* assessed using proteomics and transcriptomics

**DOI:** 10.1186/s12864-019-5456-0

**Published:** 2019-02-06

**Authors:** Camille Détrée, Iman Haddad, Emmanuelle Demey-Thomas, Joëlle Vinh, François H. Lallier, Arnaud Tanguy, Jean Mary

**Affiliations:** 1 Centro FONDAP de Investigación en Dinámica de Ecosistemas Marinos de Altas Latitudes (IDEAL), Universidad Austral de Chile, Valdivia, Chile; 2 Sorbonne Université, CNRS, Lab. Adaptation et Diversité en Milieu Marin, Team ABICE, Station Biologique de Roscoff, 29680 Roscoff, France; 3 ESPCI ParisTech, CNRS, USR 3149, Spectrométrie de Masse Biologique et Protéomique, 75231 Paris Cedex 05, France

**Keywords:** Chemoautotrophic symbiosis, Hydrothermal vent, In situ experiment, Mutualism, Proteo-transcriptomics

## Abstract

**Background:**

Colonization of deep-sea hydrothermal vents by most invertebrates was made efficient through their adaptation to a symbiotic lifestyle with chemosynthetic bacteria, the primary producers in these ecosystems. Anatomical adaptations such as the establishment of specialized cells or organs have been evidenced in numerous deep-sea invertebrates. However, very few studies detailed global inter-dependencies between host and symbionts in these ecosystems. In this study, we proposed to describe, using a proteo-transcriptomic approach, the effects of symbionts loss on the deep-sea mussel *Bathymodiolus azoricus’* molecular biology. We induced an in situ depletion of symbionts and compared the proteo-transcriptome of the gills of mussels in three conditions: symbiotic mussels (natural population), symbiont-depleted mussels and aposymbiotic mussels.

**Results:**

Global proteomic and transcriptomic results evidenced a global disruption of host machinery in aposymbiotic organisms. We observed that the total number of proteins identified decreased from 1118 in symbiotic mussels to 790 in partially depleted mussels and 761 in aposymbiotic mussels. Using microarrays we identified 4300 transcripts differentially expressed between symbiont-depleted and symbiotic mussels. Among these transcripts, 799 were found differentially expressed in aposymbiotic mussels and almost twice as many in symbiont-depleted mussels as compared to symbiotic mussels. Regarding apoptotic and immune system processes – known to be largely involved in symbiotic interactions – an overall up-regulation of associated proteins and transcripts was observed in symbiont-depleted mussels.

**Conclusion:**

Overall, our study showed a global impairment of host machinery and an activation of both the immune and apoptotic system following symbiont-depletion. One of the main assumptions is the involvement of symbiotic bacteria in the inhibition and regulation of immune and apoptotic systems. As such, symbiotic bacteria may increase their lifespan in gill cells while managing the defense of the holobiont against putative pathogens*.*

## Introduction

Symbiosis is a universal process defined as a long-lasting association between two organisms from different species; this definition encompasses the entire interaction spectrum, from mutualism to parasitism [1]. In mutualistic associations both host and symbiont(s) benefit from each other, hence promoting the adaptation of the holobiont to its environments [2]. Mutualistic associations between animals and endosymbiotic bacteria can be found in a diverse array of habitats. Most of these associations have a nutritional purpose, providing the host with essential nutrients, compounds or amino acids [3–6].

Maintaining a long-lasting mutualistic association requires the establishment of adaptive interactions from both symbionts and hosts, including fine-tuned and specific mechanisms for partners recognition, symbiosis establishment, and maintenance of symbiont densities *in hospite* [7, 8]. In the case of the host, conspicuous anatomical changes associated with the presence of symbionts are generally observed, such as the formation of specialized cells [9, 10] or organs (e.g. trophosome of the vestimentiferan tubeworm *Riftia pachyptila*) [11, 12]. Beside anatomical changes, the host immune system has been shown to be largely involved in the recognition, acceptance and regulation of symbiotic bacteria, allowing the settlement of symbionts and preventing competition with opportunistic bacteria [13, 14]. These adaptations to the symbiotic lifestyle in both hosts and symbionts have been observed among a vast array of symbiotic associations, organisms and environments, from terrestrial to deep-sea ecosystems [15–17]. Furthermore, the study of these specific symbioses may help understand the now widely recognized importance of associated microorganisms (microbiota) in virtually every organism adaptation [18].

Deep-sea hydrothermal vents are characterized by challenging physico-chemical conditions, such as total darkness and sharp gradients in temperature, pH and concentrations of reduced compounds (H_2_S, CH_4_, H_2_) [19]. Although seemingly extreme, these environments are inhabited by chemosynthetic bacteria that are capable of oxidizing these reduced compounds to produce energy and fix carbon; hence, metazoans are then able to colonize these habitats, typically via symbiotic associations with chemosynthetic bacteria [5, 20, 21].

In these symbiosis “hot spots”, a large gap has been filled in the understanding of the ecology of hydrothermal vents and physiology of associated organisms [22, 23]. In spite of all the information gathered regarding these particular associations, there have been few studies investigating the underlying molecular mechanisms involved in host-symbiont interactions.

Among the diverse organisms associated with hydrothermal vents, the mussel *Bathymodiolus azoricus* [24] constitutes an interesting model. Indeed, this bivalve from the family Mytilidae developed a highly plastic symbiosis; depending on the environmental availability of reduced compounds, it may host either one or two types of symbionts (thiotrophic and methanotrophic gammaproteobacteria) in specialized gill cells and regulate their respective densities [25–29]. *B. azoricus* is also capable of trophic plasticity; as a mixotroph, it may either use energy resources from its autotrophic symbionts or from the surrounding organic matter through regular heterotrophic filter-feeding. Indeed, unlike most other symbioses associated with hydrothermal vents, *B. azoricus* maintains a functional, though reduced, gut [30–32]. Both these symbiotic and trophic flexibilities suggest the existence of tight and specific interactions between *B. azoricus* and its endosymbionts and pave the way for possible experimental work. For instance, Kadar et al. (2005) performed a total symbiont depletion in mussels raised at atmospheric pressure, which induced a decrease in their fitness [33]. More recently, a metabolic interdependence between *B. azoricus* and its symbionts has been evidenced using a proteo-genomic approach comparing aposymbiotic and symbiotic tissues [34]. While none of these studies have been validated in situ, it is noteworthy that pioneering attempts to do so have shown the putative role of two families of immune proteins in symbionts’ recognition and regulation [35, 36]. Although significant in the understanding of symbiotic association in deep-sea environments, these studies were either performed ex situ or limited to specific processes or families of genes, restricting our understanding on the mechanisms established by the host to manage its symbionts.

In this context, and to gain a better understanding on symbiotic interactions in deep-sea hydrothermal vents, the objective of this study was to generate a comprehensive proteomic and transcriptomic overview of the mussel *B. azoricus’* response to symbiont-depletion. In order to detect host’s proteins and genes involved in symbiotic functions, a depuration of symbionts was conducted in situ and the gill proteome and transcriptome of mussels were compared between control and symbiont-depleted individuals. To gain an insight into the adaptive features established by *B. azoricus* to specifically recognize and manage its symbionts, we first hypothesized that both immune and apoptotic pathways may be relatively over-represented in symbiont-depleted mussels, as these processes have been shown to be primordial in the regulation of symbionts in model symbioses. Finally, we hypothesized that in situ symbiont depletion may impact mussels’ energetic metabolism, and hence, investigated the effects of symbiont depletion on mussels’ carbon metabolic process in order to appraise the nutritional dependency of *B. azoricus* to its symbionts.

## Results

### Global changes at the proteome level

**Fig. 1:**
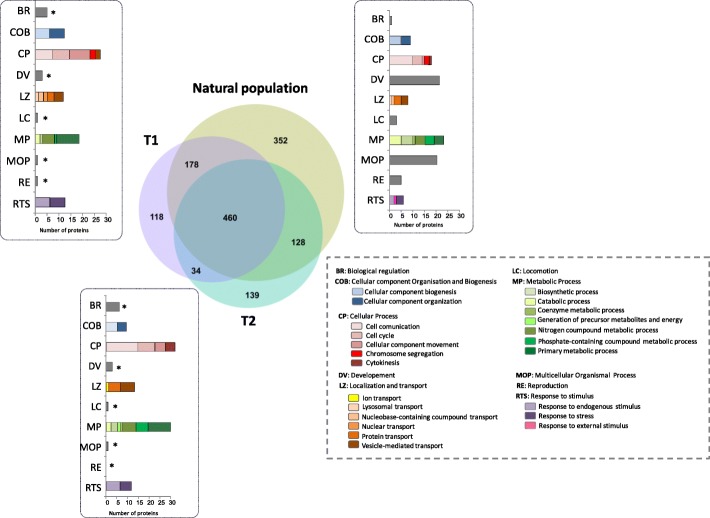
Venn diagram of the identified proteins in each condition (Natural population, T1, T2) and functional annotation. Numbers inside circles represent the number of exclusive proteins and numbers inside the overlapping areas represents shared proteins. Functional annotation of biological processes was performed on proteins exclusive to each condition and according to Gene Ontology (GO).*: Significantly different from the same process in natural population

LC-MS/MS analyses led to the identification of a total of 1409 proteins with at least two unique peptides, in our three conditions: symbiotic mussels (natural population), mussels partially depleted in symbionts (T1) and aposymbiotic mussels (T2). A diminution of the total number of proteins identified was observed throughout the symbiont-depletion experiment, with a total of 1118 in symbiotic mussels, 790 in partially depleted mussels and 761 in aposymbiotic mussels (Fig. 1). While symbiotic and depleted mussels shared a high number of identified proteins (58 and 60% with T1 and T2 respectively), the diminution observed may rather be associated with the decrease of unique proteins, from 352 in symbiotic animals to less than 140 in symbiont-depleted conditions. Genes Ontology (GO) analyses of these proteins solely identified in one of the three conditions revealed their implication in fundamental biological processes with distinct repartition according to symbiotic condition (Fig. 1). In symbiotic individuals most of the proteins identified were involved in four main biological processes: development (19%), multicellular organismal process (23%), metabolic process (22%) and cellular process (15%). Following symbionts’ depletion, several proteins were also involved in metabolic process (between 20 and 25% in T1 and T2) and cellular process (between 25 and 30% in T1 and T2), but a significant decrease – in comparison with individuals with natural population – of the number of protein involved in development (5% T1 and 3% T2) or multicellular organismal (1 and 2% for T1 and T2 respectively) was observed. In depleted conditions, proteins were rather associated with localization process (12–15%), cellular component organization and biogenesis (10–14%) or response to stimuli (10–12%). It is noteworthy that proteins involved in cellular processes were more abundant after symbiont-depletion, especially proteins involved in cell communication, cell cycle and cellular component movement. In partially depleted and aposymbiotic mussels, a greater number of proteins associated with ion, proteins and vesicle-mediated transport was observed as compared to symbiotic organisms. In addition, the number of proteins associated with reproduction and development decreased dramatically with symbionts depletion. Finally, congruent augmentation of stress related-proteins and diminution of proteins responding to external stimuli were observed after both partial and complete loss of symbionts. In these conditions, proteins were rather involved in the response to endogenous stimuli.

**Fig. 2:**
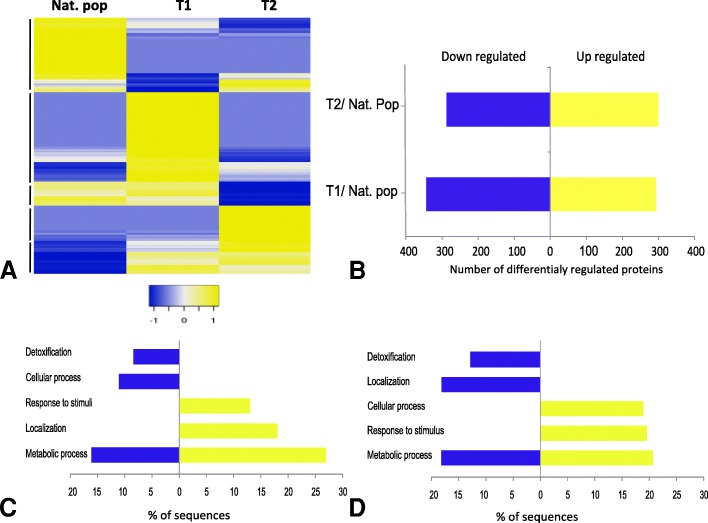
Expression and biological functions of proteins shared between symbiotic (Nat. pop), partially depleted (T1) and aposymbiotic mussels (T2). **a** Heatmap depicting clustering of proteins in the three conditions, black bars on the left side indicate clusters of protein, **b** Number of differentially concentrated proteins in partially depleted (T1) and aposymbiotic mussels (T2) compared to symbiotic mussels (Nat. pop) (Fold change > 1.5), **c** Five most represented biological process with which differentially concentrated proteins were associated in partially depleted mussels (T1) and **d** in aposymbiotic mussels (T2)

To further identify proteins that may be involved in host-symbionts interactions, a quantification of shared proteins was performed and showed distinct concentration patterns in proteins identified in symbiotic, partially depleted and aposymbiotic mussels (Fig. 2a). Overall, when compared to symbiotic animals, 344 and 288 proteins were found down-regulated in T1 and T2, respectively (Fig. 2b). These down-regulated proteins were mostly involved in detoxification and metabolic processes after symbionts’ depletion (T1 and T2), but also in cellular processes in the case of partial depletion or localization in aposymbiotic animals (Fig. 2c and d). 294 proteins were found up-regulated after a partial depletion of symbionts (in comparison with symbiotic animals) and were mainly associated with the response to stimuli, localization and metabolic processes. The 300 proteins up-regulated in aposymbiotic animals were mainly involved in cellular processes, response to stimuli and metabolic processes (Fig. 2b, c, d).

### Global changes at the transcriptome level

**Fig. 3:**
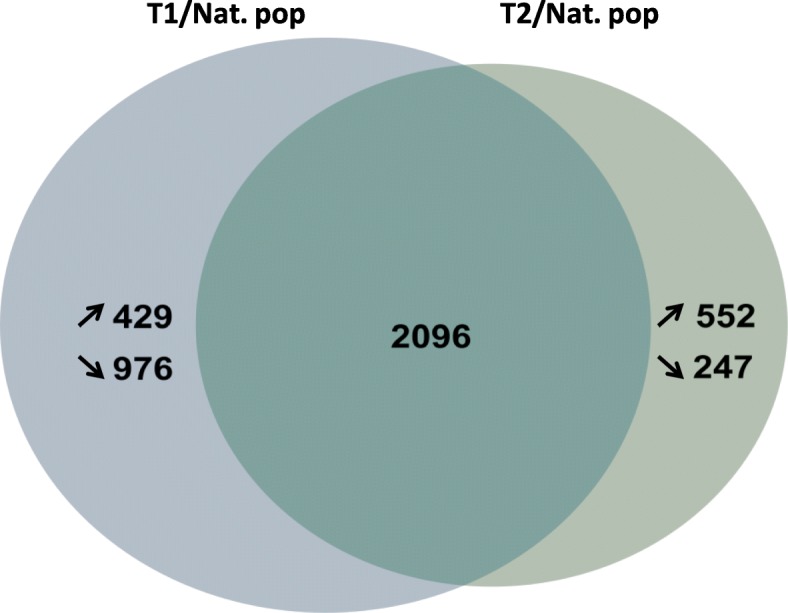
Venn diagram of differentially expressed genes (DEG) comparing partially depleted (T1) vs symbiotic mussels (Nat. pop) and aposymbiotic (T2) vs symbiotic mussels (Nat. pop). Numbers in the middle of circles represent the number of DEG exclusive to a comparison while the number inside the overlapping area represents shared DEG.

**Fig. 4:**
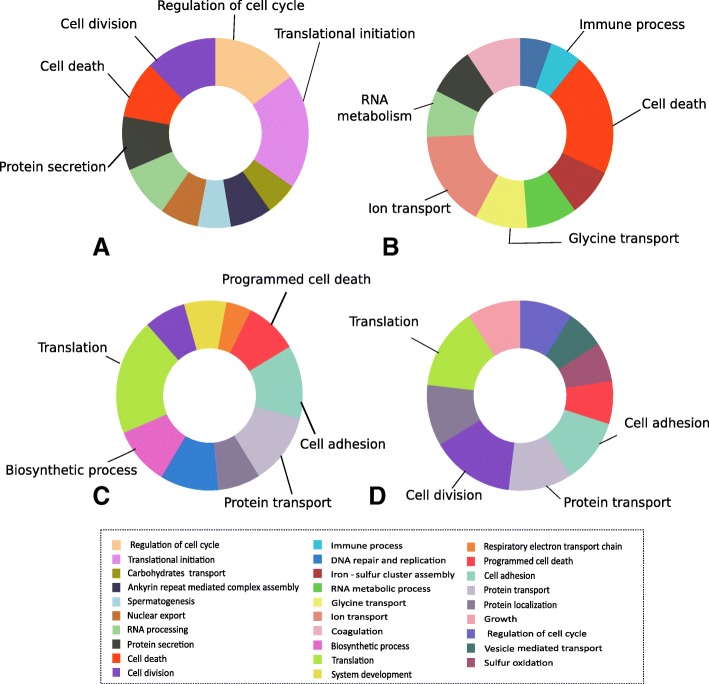
Functional classification of DEGs based on the GO biological process associated terms. **a** Functional classification of genes up-regulated in partially depleted mussels (T1) and **b** aposymbiotic mussels (T2), in comparison to symbiotic organisms and **c** functional classification of down-regulated genes in partially depleted mussels (T1) and **d** in aposymbiotic mussels. Biological processes labeled in the figure are the one discussed in the text

Global transcriptomic analyses using microarray allowed the identification of 4300 transcripts differentially expressed between symbiont-depleted (T1 or T2) and symbiotic mussels. Among these transcripts, 799 were found differentially expressed only in aposymbiotic mussels and almost twice as many (1405) were found differentially expressed only in partially-depleted mussels as compared to symbiotic mussels (Fig. 3). A gene ontology (GO) analysis of differentially expressed genes (DEG) was performed to assess which pathways were differentially regulated between the three conditions. GO analysis revealed that up-regulated genes in partially depleted-mussels were mostly involved in the regulation of translational initiation (19%) and cell cycle (15%) but also in cell death and division, and proteins secretion (Fig. 4a). Down-regulated genes were involved mostly in translation (20%), cell adhesion (13%) or protein transport (12%), but also in biosynthetic process and programmed cell death (Fig. 4c). In aposymbiotic mussels, up-regulated genes were mostly associated with cell death (21%) and ion transport (17%) and other processes such as glycine transport, RNA metabolism or immune processes (Fig. 4b) while down-regulated genes were involved mainly in translation (14%), cell division (15%) along with cell adhesion and protein transport (Fig. 4d).

### Global activation of immune-related processes in symbiont-depleted mussels as compared to symbiotic mussels

Symbionts depletion led to the disruption of mussels’ immune system, evidenced at both transcriptome and proteome level, by a global immuno-stimulation. This increase in immune-related proteins is less pronounced in partially depleted individuals, where only three proteins – mainly involved in the activation of the NF-KB cascade (Mitogen-activated protein kinase 14, Myeloid differentiation primary response protein 88, Dual specificity mitogen-activated protein kinase kinase 1) – were found to be up-regulated while two proteins were down-regulated (PGRP 3, Complement c1q-like protein 4) (Table 1). Surprisingly, in this same condition, the PeptidoGlycan Recognition Protein 3 (PGRP 3) which is a pattern recognition receptor (PRR), involved in the recognition of non-self, was found up-regulated at the transcript level. Other PRR such as the Toll-Like Receptors (TLR) 1 and 13 and the TNF ligand superfamily member 10 were also up-regulated at the transcript level in partially depleted organisms (Tables 1 and 2). In aposymbiotic mussels, both transcript and protein of the PGRP3 were found down-regulated while several PRRs transcripts were found up-regulated (TLR-1, TLR-13, TLR 20a C-type lectins domain family 4, 6 and 7, IL-17). In this same condition, transcripts involved in immune signal transduction (Mitogen-activated protein kinase kinase kinase 4, Mitogen-activated protein kinase kinase kinase kinase 5, Myeloid differentiation primary response protein 88) were found up-regulated. In addition, in both conditions, only two immune-effectors (Lysozyme 3 and the antimicrobial peptide Hydramacyn) were found differentially expressed compared to symbiotic condition. For the lysozyme 3, both transcript and protein were found up-regulated in aposymbiotic mussels while in the case of hydramacyn, a down-regulation at the transcript level was observed in both symbiont depleted and aposymbiotic organisms.

**Table 1:** Overview of proteins and transcripts differentially regulated between natural population and symbionts-depleted conditions and involved in immune related pathways

ID	T1		T2	
	*P*	T	*P*	T
TOLL/NF-KB cascade				
Mitogen-activated protein kinase 14	**+**	**/**	**+**	**/**
Mitogen-activated protein kinase kinase kinase 4	**/**	**/**	**/**	**+**
Mitogen-activated protein kinase kinase kinase kinase 5	**/**	**–**	**/**	**+**
Myeloid differentiation primary response protein 88	**+**	**+**	**/**	**+**
Dual specificity mitogen-activated protein kinase kinase 1	**+**	**/**	**/**	**/**
TLR 20 a	**/**	**/**	**/**	**+**
TLR 2	**/**	**–**	**/**	**/**
TLR 1	**/**	**+**	**/**	**+**
TLR 13	**/**	**+**	**/**	**+**
Toll-like receptor 6	**/**	**–**	**/**	**/**
Nuclear factor Nf-kappa-b p105 subunit isoform 2	**/**	**/**	**/**	**+**
Nf-kappa-b inhibitor-interacting protein	**/**	**–**	**/**	**+**
Others				
Fibrinogen c domain binding prot 1 like	**/**	**+**	**/**	**+**
Suppressor of cytokine signaling	**/**	**–**	**/**	**–**
Antimicrobial peptide hydramacin	**/**	**–**	**/**	**–**
Lipopolysaccharide-induced tnf-alpha factor	**/**	**+**	**/**	**+**
PGRP 3	**–**	**+**	**–**	**–**
Complement c1q-like protein 4	**–**	**+**	**/**	**+**
Complement c1q tumor necrosis factor-related protein 2	**/**	**+**	**/**	**+**
Complement c1q tumor necrosis factor-related protein 3-like	**/**	**+**	**+**	**+**
Complement c1q subcomponent subunit b	**/**	**+**	**/**	**+**
Complement c1q-like protein 3	**/**	**/**	**/**	**+**
C1q domain containing protein 1q2	**/**	**/**	**+**	**/**
Complement component 1 q subcomponent-binding mitochondrial-like	**/**	**/**	**–**	**/**
Lysozyme 3	**/**	**/**	**+**	**+**
Macrophage migration inhibitory factor	**/**	**/**	**–**	**+**
C-type lectin domain family 4 member k	**/**	**/**	**/**	**+**
C-type lectin domain family 6 member a	**/**	**/**	**+**	**+**
C-type lectin domain family 7 member a isoform 7	**/**	**+**	**/**	**+**
Collectin-12	**/**	**/**	**/**	**+**
Interleukin 17-like	**/**	**/**	**/**	**+**

P Proteins, T Transcripts, +: up-regulated compared to the natural population, −: Down-regulated, /: No significant differences

**Table 2:** Overview of proteins and transcripts differentially regulated between natural population and symbionts-depleted conditions and involved in programmed cell death

ID	T1		T2	
	*P*	T	*P*	T
Effector caspases				
Caspase 3 b	/	+	/	+
Caspase 3	/	+	+	+
Caspase-3 precursor	–	–	–	/
Initiator caspases				
Caspase partial	–	/	/	+
Caspase-10-like	/	/	/	+
Caspase 8	+	+	/	+
Initiator caspase	/	–	/	+
Caspases activators				
TNF alpha	/	+	+	+
TNF ligand superfamily member 10	/	+	/	+
TNF ligand superfamily member 11	/	–	/	–
Fas ligand	/	/	+	+
Fas associated factor 1	/	–	/	/
Fas-associated factor 2	/	+	/	+
Death domain-containing protein cradd	–	/	–	/
Death-associated protein 1-like	+	/	+	+
Programmed cell death protein 4	+	+	/	+
Programmed cell death protein 4-like	/	+	/	+
Programmed cell death protein 5	/	+	+	+
Programmed cell death 6 interacting protein	/	–	/	–
Translationally-controlled tumor	–	/	/	/
Apoptosis inhibitors				
Inhibitor of apoptosis protein	/	+	/	+
Apoptosis 1 inhibitor	/	–	/	–

P Proteins, T Transcripts, +: up-regulated compared to the natural population, −: Down-regulated, /: No significant differences

### Activation of programmed cell death mechanism in symbiont-depleted mussels in comparison to symbiotic mussels

Similarly to the activation of immune process, activation of programmed cell death seemed to occur mainly in aposymbiotic mussels (less clearly in partially depleted mussels). This activation was first evidenced by the up-regulation at both protein and transcript level of several activators of apoptosis such as cell death proteins (e.g. Death-associated protein 1-like, Programmed cell death protein 4 and 5) but also ligand and proteins associated with TNF receptors (TNF alpha, Fas ligand, Fas associated factor 2, TNF ligand superfamily member 10) (Table 2). In addition, the up-regulation of the initiator caspases transcripts (e.g. Caspase 8, Caspase 10-like, Initiator caspase) transcript and the executioner Caspase 3 (at both transcript and protein level) in aposymbiotic mussels evidenced an overall initiation of cellular component degradation. In partially depleted mussels, apoptotic response is more contrasted. For instance, at the protein level, initiator caspases were found concurrently up and down-regulated (Caspase 8 and Caspase partial, respectively) and a similar contrast was observed for caspases activators (Death-associated protein 1-like, Programmed cell death protein 4 up-regulated and Death domain-containing protein Cradd, Translationally-controlled tumor isoform 1 down-regulated). Similarly, at the transcriptome level, we observed simultaneously the up-regulation of the executioner Caspase 3 and 3b, the down-regulation of Caspase-3 precursor, all involved in the degradation of cellular components.

### Disruption of carbon metabolic process in partially depleted and aposymbiotic mussels

Animals that experienced a partial or almost complete depletion of symbionts showed a disruption of carbon metabolism, and this at both protein and transcripts levels. First, the formation of pyruvate via the glycolysis metabolic pathway was found to be affected by symbionts depletion. After partial symbiont-depletion, 3 key enzymes involved in the sixth, seventh and final step of glycolysis were found down-regulated at the protein level (Glyceraldehyde 3-Phosphate dehydrogenase, Phosphoglycerate kinase and Pyruvate kinase) (Table 3). Quite oppositely, the Mannose-6-phosphate isomerase (catalyzing the isomerization of mannose-6-phosphate to fructose-6-phosphate) was found up-regulated at the protein level. At the transcript level, and contrary to what has been observed at the proteome level, both G3P dehydrogenase and Pyruvate dehydrogenase (catalyzing the transformation of pyruvate into Acetyl-CoA) were found up-regulated. Pyruvate dehydrogenase was also found down-regulated at the transcriptome level in aposymbiotic mussels, along with enzymes catalyzing the first (Hexokinase 2) and sixth (G3P dehydrogenase) steps of the glycolysis metabolic pathway. Results at the proteome level are more contrasted: we observed both the down-regulation of the enzyme catalyzing the final step of glycolysis and the up-regulation of enzymes catalyzing the sixth and eighth steps (G3P dehydrogenase and Phosphoglucose isomerase).

**Table 3:** Overview of proteins and transcripts differentially regulated between natural population and symbionts-depleted conditions and involved in main carbon metabolism pathways

ID	EC number	T1		T2	
		*P*	T	*P*	T
Pentose phosphate pathway					
Glucose-6- Phosphate dehydrogenase	1.1.1.49	–	**/**	–	/
Transaldolase	2.2.1.2	–	**/**	–	/
Transkelotase like protein 2	2.2.1.1	–	**/**	/	/
Deoxyribose Phosphate aldolase	4.1.2.4	+	**/**	+	/
Glycolysis					
Phosphoglycerate kinase	2.7.2.3	–	**–**	/	/
Pyruvate kinase	2.7.1.40	–	**/**	–	/
Phosphoglucose isomerase	5.3.1.9	/	**/**	+	/
Glyceraldehyde-3-Phosphate dehydrogenase	1.2.1.13	–	**+**	+	–
Mannose-6-phosphate isomerase	5.3.1.8	+	**/**	/	/
Pyruvate dehydrogenase	2.3.1.12	/	**+**	+	–
Hexokinase type 2	2.7.1.1	/	**/**	/	–
TCA Cycle					
2-oxoglutarate dehydrogenase	2.3.1.61	–	**–**	/	/
2-oxoglutarate/malate carrier protein	/	–	**/**	–	/
Succinate dehydrogenase	1.3.5.1	–	**–**	–	/
Malate dehydrogenase	1.1.1.37	/	**–**	–	–
Isocitrate dehydrogenase	1.1.1.42	–	**–**	–	**–**
Aconitate hydratase	4.2.1.3	/	**–**	–	**/**
ATP citrate synthase	2.3.3.8	/	**/**	**+**	**/**
Others					
Glycerol-3- phosphate dehydrogenase	1.1.1.8	–	**–**	**–**	**–**
Pyruvate carboxylase	6.4.1.1	/	**–**	**/**	**–**

P Proteins, T Transcripts, +: up-regulated compared to the natural population, −: Down-regulated, /: No significant differences

Although Glycolysis and Pentose phosphate pathways may occur concurrently in cells, using the G6P, we observed no alterations of the pentose phosphate pathway at the transcript level in symbiont-depleted organisms (Table 3). In contrast, at the protein level, a global down-regulation of several enzymes was observed in all the symbiont-depleted conditions. These proteins were involved in the production of the 6-phosphogluconolactone, the fructose 6-phosphate and erythrose 4-phosphate or in the production of ribulose-5-phosphate (Ru5P). Despite the global down-regulation observed at the protein level, it is worth noting that the deoxyribose-phosphate aldolase – involved in the transformation between the 2-deoxy-D-ribose 5-phosphate and the D-glyceraldehyde 3-phosphate – was found up-regulated in all symbiont-depleted conditions.

Finally, key enzymes of the Citric acid cycle (TCA cycle) were also found differentially regulated after symbiont depletion (Table 3). In T1, after a partial symbiont loss, the 2-oxoglutarate dehydrogenase (involved in the transformation of 2-oxoglutarate into succinyl-CoA) and the Succinate dehydrogenase (catalyzing the oxidation of succinate to fumarate) were found down-regulated at both protein and transcript level. At the transcriptomic level, the Isocitrate dehydrogenase and Aconitase hydratase, two enzymes involved in the first reactions of the TCA cycle and in the production of α-ketoglutarate, were found down-regulated along with the Malate dehydrogenase that catalyzes the oxidation of malate to oxaloacetate. Interestingly, these three enzymes were also found down-regulated in aposymbiotic mussels, at the protein level – along with the succinate dehydrogenase – while only the Malate dehydrogenase and Isocitrate dehydrogenase were down-regulated at the transcript level.

### Degree of congruency between LC-MS/MS and microarray

Irrespective of the pathway, a low degree of congruency was observed between protein concentration and mRNA level (from 3 to 27%) (Table 4). In the case of immune process and programmed cell death, very few of the differentially concentrated protein were found associated with DEG (respectively 1 and 3 association) for T1 whereas for T2 congruency between protein concentration and gene expression was higher (respectively 4 and 5 associations). For metabolic processes, an opposite response was observed with a higher percentage of congruency in T1 than in T2. Despite these observed differences, no significant differences were observed between T1 and T2 irrespective of the pathways.

**Table 4:** Congruency analyses between protein concentration and mRNA level

	Treatment	Congruency (%)	Fishers’ exact test
Immunity	T1	3	*p* > 0.05
	T2	13	
Apoptosis	T1	14	*p* > 0.05
	T2	27	
Metabolism	T1	25	*p* > 0.05
	T2	15	
Total	T1	10	*p* > 0.05
	T2	15	

## Discussion

The symbiosis between the deep-sea mussel *B. azoricus* and its endosymbionts provides an interesting case study to deepen our knowledge on the molecular mechanisms at work in symbiotic interactions. In the present study, the in situ depletion of symbionts led to a global dysregulation – at both transcript and protein levels – of host machinery, impacting crucial process such as translation, cell cycle and division or transport. Furthermore, the activation of both immune system and programmed cell death processes in symbiont-depleted mussels suggest a fundamental role of these processes in adaptation to symbiotic lifestyle. In addition, the important modulation of enzymes involved in carbon metabolism suggests a putative nutritional dependency of *B. azoricus* on its symbionts, in spite of its ability to rely on particulate organic matter by filter-feeding.

Although traditionally thought to be solely involved in defense against pathogens, host immune system has been shown to be one of the key features allowing the establishment of fine-tuned associations between host and symbionts in metazoans/microbes mutualistic association [13, 14]. *B. azoricus*’ symbiotic tissue display an extensive and complex immune collection [37, 38] which was, in the first place, perceived as a tool to maintain symbionts under control inside the gills [37, 39]. However, our results may evidence an opposite mode of action: the increased expression of immune-related genes in both symbiont-depleted conditions, even more pronounced in aposymbiotic mussels, may rather evidence an activation of host immune system in the absence of symbionts. In partially depleted mussels, the activation at the transcriptome level of several Toll like receptors (TLRs 1, 3) and proteins involved in the transduction of TLRs signal could indicate a response to microorganism invasion. Indeed, Toll-like receptors are a family of PRRs involved in the recognition of pathogenic microorganisms, and may trigger the activation of an adjusted defense response via the activation of a cascade of proteins until production of immune executioners [40]. Interestingly, the number of up-regulated PRRs such as TLRs, C-types Lectin or Collectin increased with symbiont-depletion. From these results, two main hypotheses could be formulated. Owing to the key role of PRRs in the establishment of defense mechanisms, we can hypothesize that when symbionts are no longer essential for host nutritional input – due for instance to the limited availability of the reduced compounds – they may no longer be welcome and will rather be perceived as pathogens by the host, hence inducing the activation of host defense system. Alternatively, considering the important role of thiotrophic symbionts in host defense against pathogens [41] the loss of endosymbionts may force the host to take over and trigger its own defense system. The up-regulation of most of the PRRs at transcripts level only, supports this second hypothesis: mussels may have lined up storage of immune transcripts to respond adequately to a putative pathogen invasion. In contrast, the global down-regulation of another immune receptor (PGRP 3), seems to ascertain its role as a symbiont recruiter [36].

Concurrently to the activation of host immune system, activation of programmed cell death occurred mainly in aposymbiotic mussels (less clearly in partially depleted mussels). Apoptosis or programmed cell death-I is responsible for the maintenance of organisms’ homeostasis via the elimination of infected or unnecessary cells, or simply via their constant renewal [42]. In our study, the activation of the extrinsic apoptotic pathways evidenced in aposymbiotic mussels, by the up-regulation of both death receptor ligands (TNF α and Fas ligand) and the executioner Caspase 3, may suggest an elimination of gill cells that do no longer contain alive or effective symbionts. However, these results may also suggest that in symbiotic mussels, symbionts may manipulate and block host cells’ apoptosis to increase their life span within gill cells or to spread in other cells as observed in parasitic associations [42].

Metabolically, the in situ depletion of symbionts in *B. azoricus* gills induced, after 7 days, a down-regulation of several enzymes involved in the energy yielded phases of the glycolytic pathway. The glycolytic pathway allows the oxidation of glucose into pyruvate which can be transported to the mitochondria and converted into acetyl-CoA that will in turn feed the TCA cycle and lead to the production of cofactors and ATP to fuel oxidative phosphorylation [43, 44]. The observed decrease in expression of glycolytic enzymes may thus have led to a lower production of Acetyl-CoA, which may explain the observed down-regulation of TCA cycle enzymes. These results, combined with the down-regulation of enzymes from the pentose phosphate pathway, suggest an overall metabolic depression during the first days of symbiont-depletion, which may be mainly due to a drastic diminution of nutrient intakes. In aposymbiotic mussels, the up-regulation of enzymes of the glycolytic pathways could suggest a renewal of nutrient input via symbionts’ digestion. In this case, the glucose entering the cell could be used in both glycolysis and sorbitol pathways, bypassing both the TCA cycle and the pentose phosphate pathway, to finally feed the lipogenesis and provide energy reserves when food is limiting [45]. However, none of the enzymes specifically involved in the sorbitol or lipogenesis pathway was found differentially regulated in depleted mussels. An alternative hypothesis could be brought by a recent proteo-genomic study on *B. azoricus*’ symbionts which showed that thiotrophic symbionts may not be able to produce several TCA cycle enzymes [34]. The authors suggest that the missing enzymes (Malate dehydrogenase, Succinate dehydrogenase) may be provided by the host. Interestingly, these two enzymes were herein down-regulated either in partially or fully depleted mussels suggesting that the global down-regulation of TCA cycle enzymes may reflect the depletion of symbionts without necessarily indicating a metabolic shift. Nevertheless, one of the main assumptions is that partially depleted mussels were relying solely on nutrient-provisioning endosymbionts whereas aposymbiotic mussels mainly relied on the digestion of symbionts [28, 35]. Although the switch to a filter-feeding mode cannot be excluded, the up-regulation in the gills of Lysozyme 3, involved in the degradation of bacterial cell wall, strengthen the hypothesis of symbiont digestion in aposymbiotic mussels.

Finally, among the 73 genes and proteins studied, only 8 and 11 (in symbiont-depleted and aposymbitic mussels respectively) differentially concentrated proteins were associated with DEG (10 and 15% respectively). Low correlation or congruency between protein concentration and gene expression has already been documented in yeast [46], plants [47], and mammals [48], but also in the symbiotic associations between the corals *Pocillopora acuta* or *Seriatopora hystrix* and their *Symbiodinium* [49, 50]*.* Such differences between mRNA levels and protein concentration can be explained biologically by distinct turnover of proteins and transcripts and/or the numerous posttranslational modifications in proteins. Furthermore, the divergence between protein concentration and mRNA level may also be due to the lower sensitivity of the proteomic analysis.

## Conclusion

Overall, our results confirm a significant reliance of *B. azoricus* on its symbionts, which could be perceived as an adaptive feature to symbiotic lifestyle. Our results uphold the nutritional inter-dependency already observed ex situ but also suggest that symbiotic bacteria may be involved in the inhibition and regulation of immune and apoptotic systems. As such, symbiotic bacteria may increase their lifespan in gill cells while managing the defense of the holobiont against putative pathogens*.* However, our data do not allow us to disentangle between this hypothesis and a simpler explanation in which the drastic diminution of symbiont population compelled *B. azoricus* to produce its own defense, and to wipe bacteriocytes out. Further in situ studies will be needed to scrutinize these possible mechanisms. As for now, our in situ study provides a solid proteo-transcriptomics basis of the response of the deep-sea mussel *B. azoricus* to symbiont variations.

## Methods

### Animal collection: Field sampling and in situ experiments

Sampling and in situ experiments were conducted at one hydrothermal vent site (Lucky Strike Montsegur, 37°17’ N, 32°17’ W 1700 m depth), located on the Mid Atlantic Ridge, during the BioBaz [51] and MoMARSAT [52] cruises in July and August 2013. Physico-chemical parameters of this vent site have been extensively studied and well characterized [53, 54]. At the beginning, about 30 specimens of the deep-sea mussel *Bathymodiolus azoricus* were collected within a mussel bed in the vicinity of the hydrothermal fluid (chimneys) using the Remote Operating Vehicle (ROV), Victor 6000, remotely controlled from the Research Vessel “Pourquoi pas?”. These individuals - further referred to as “natural population”, corresponding to the first time (T0) of the in situ experiment – were then dissected to sample their gills, which were frozen in nitrogen, and stored at − 80 °C until further analyses. Subsequently, an in situ depletion of symbionts has been performed through a translocation of 30–40 individuals, divided in two wire steel cages, a dozen meters away from the hydrothermal fluid, capitalizing on a natural gradient in concentration of reduced compounds (H_2_S, CH_4_) between the mussel bed and the translocated site. The recovery of these cages was performed after 7 and 27 days (respectively T1 and T2) after the start of the translocation. Immediately after collection, gills were dissected, frozen in liquid nitrogen and stored at − 80 °C until further analysis. The relative quantification of thiotrophic symbionts (SOX) hosted in *B. azoricus’* gills – measured by qPCR – indicated reductions of 50 and 90% of symbiotic content at T1 and T2 respectively [35]. In the case of methanotrophic bacteria (MOX), an almost null abundance was measured in individuals from natural population and MOX were no longer detectable by qPCR (below detection range) after in situ experiments. Individual from the natural population (T0) will be referred hereafter as symbiotic mussels, while individuals with a reduction of 50% (T1) will be referred as partially-depleted mussels and individuals with a 90% reduction (T2) will be referred as aposymbiotic mussels. It is noteworthy that no deterioration of body condition or gill structures was observed in sampled individuals.

## Proteomic approach

### Proteins extraction and separation

Gill tissues were ground in a precooled mortar in the presence of liquid nitrogen and protein extraction was performed using the TCA/acetone method according to Mechin et al. (2007) [55]. For each condition, a pool of proteins from 6 individuals (75 μg of total protein) [56], was separated on gels (a 12% acrylamide/bisacrylamide gel (Laemmli, 1970)) using a BioRad Mini-PROTEAN® system at a constant intensity of 40 mA for about 1 h. Proteins were then visualized with Brillant Blue G-Colloidal (Sigma, Saint-Louis, Missouri) according to manufacturer’s instructions. After coloration, each track of the gel was cut in fifteen bands.

### Tryptic digestion

In-gel tryptic digestion method was used on the purified samples as described by Shevchenko (2006) [57]. Briefly, after reduction–alkylation (5 mM dithiothreitol in 50 mM NH_4_HCO_3_, 30 min at 56 °C; 25 mM iodoacetamide in 50 mM NH_4_HCO_3_, 20 min in dark at room temperature), proteins in gel pieces were digested by incubation with 12.5 ng.μL^− 1^ Trypsin (modified sequencing grade, Roche) in sodium carbonate, overnight at 37 °C with gentle shaking. The reaction was stopped with one reaction volume (50 μL) 5% formic acid. Subsamples were sonicated for 10 min in an ultrasonic bath at room temperature and desalted using C18 packed column (C18 ZipTip®, Millipore, Billerica, MA, USA) prior to nano LC-MS/MS analyses.

### MS/MS analyses

For each analysis, 8 μL of the peptide solution was loaded on a precolumn (C18 Acclaim PepMap 100 Å, 1 mm id, 500 mm length, Dionex) and eluted on a capillary reversed phase column (nano C18 Acclaim PepMap100 Å, 75 μm i.d., 50 cm length, Dionex), at a constant flow rate of 220 nL/min, with a gradient 2 to 50% buffer B during 170 min followed by 50 to 60% during 10 min (buffer A: water/ACN/FA 98:2:0.1 (*v*/v/v); buffer B: water/ACN/FA 10:90:0.1 (v/v/v)). The MS analysis was performed on a FT ICR mass spectrometer (LTQ-FT Ultra, ThermoFisher Scientific, San Jose, CA) with the top 7 acquisition method: MS resolution 60,000, mass range m/z 500–2000, followed by 7 MS/MS (LTQ) on the 7 most intense peaks, with a dynamic exclusion of 90 s. The raw data were processed using Proteome Discover v.1.4 (ThermoFisher Scientific) and Mascot v.2.5 (Matrix Science). Each sub-sample was analysed in triplicate. The database search was done on merged data using Mascot search engine on all taxa using SwissProt database (version 2014_06, 53,6489 sequences) and *B*. *azoricus* libraries [58]. The following parameters were used: up to 2 miss-cleavages; MS tolerance 10 ppm; MS/MS tolerance 500 mma; full tryptic peptides; partial modifications carbamidomethylation (C), oxidation (M). Validation on DAT files (from Mascot results) was performed on proteins using MyproMS software [59]. Proteins were selected when at least two peptides with a minimum score of 25 were identified. The relative quantification of the proteins was done using Maxquant software version 1.5.2.8 according to Cox et al. (2014) [60]. Briefly, peptide lists were searched against the Uniprot/SwissProt database (version 2014_06) using the Andromeda search engine [61] with the same parameters used previously in Mascot: two missed-cleavages were allowed, protease configuration set as C-terminal to arginine and lysine, carbamidomethylation (C) as a fixed modification and oxidation as a variable modification with a cut-off FDR of 0.01. Bioinformatics analyses to compare proteins expression between symbiotic and symbiont-depleted condition were performed using the Perseus software of the Maxquant platform [62]. Technical replicate of protein expression values were averaged and log2-transformed. To visualize expressional differences, a hierarchical clustering of expression values was conducted according to Pearson distance (http://heatmapper.ca).

## Transcriptomic approach

### Array construction

A custom heterologous 8X105K 60-mer oligonucleotide array has been produced using the Agilente Array application (https://earray.chem.agilent.com/earray/) including 61,300 unique cDNA expressed sequence tag (EST) and 27,062 annotated probes corresponding to around 13,000 unique genes. All sequences used have been obtained from previous sequencing of *B. azoricus* transcriptome (see [37]; Read Archive under the accession n° SRA024338 and additional RNAseq sequencing (Sequence Read Archive (DRA, http://trace.ddbj.nig.ac.jp/dra/) with accession number DRA 004082). Probes were synthetized with positive and negative control using 8X60K-feature Agilent slide format. Probe sequences are available at the gene expression omnibus database (https://www.ncbi.nlm.nih.gov/geo/) under the accession number GSE124699.

### Array hydridization

Total RNA was extracted from individual gills of *B. azoricus* from the natural population and from each in situ experiment condition using TRIzol reagent (Sigma-Aldrich, USA). Following extraction, RNA concentration purity and integrity were checked. Two pools containing 40 ng of total RNA extracted samples from T1 and T2 conditions (5 samples per pool, 2 pools per condition) and four pools (6 individuals per pool) for T0 were prepared. Labeled Cy3 or Cy5 probes were synthetized from 200 ng of total RNA using Agilent’s Low Input Quick Amp labelling Kit following manufacturer’s protocol. Labelled probes were then purified using Kit Illustra Cy Scribe GFx purification (GE HealthCare) and both quantity and quality were determined using NanoDropND-1000 spectrophotometer (Nanodrop Technologies, Delaware, USA). For microarray hybridization 800 ng of probes were firstly fragmented (60 °C/30 min) using fragmentation buffer (Agilent Technologies, USA) and then added to 25 μL of 2X hybridization buffer (Agilent Technologies, USA). Arrays were hybridized in chambers (Appligen, USA) (65 °C/17 h). Following hybridization, arrays were washed twice with buffer-1 containing (500 µL Agilent Technologies, IncWilnington, USA and 250 µL Triton 10X, 1 min), once with buffer-2 containing (250 µL Agilent Technologies, IncWilnington, USA and 250 µL Triton 10X, 1 min) at 37 °C and finally once with acetonitrile (200 mL, 10 s). Hybridized microarrays were scanned with an Agilent scanner using the Feature Extraction Software (version 9.5.3.1, Agilent Technologies, Inc). This process was repeated for all the hybridized slides with a 5-μm resolution mode. Data from each probe was then extracted and analysed.

### Statistical analyses on microarray data

The microarray data were analysed as described earlier [25] using both the statistical language R Studio (http://www.rstudio.com) and BioConductor [63]. Limma library [64] was used for the analysis and comprehension of high-throughput data and the linear model for microarray data. The background correction of the probe intensity was carried out using the normexp method. Technical artefacts were removed using quantile normalization while intra- and inter-slide loess normalizations were applied to remove intensity dependent trends. Only probes showing signal intensity three times superior to the background signal – and this, in more than 20% of the samples – were kept for the analysis. Intensities’ data were then log2-transformed and replicated values of each gene were averaged. The expression ratio of each gene was then calculated by dividing the log_2_ signal intensity of the gene for one given sample by the average log_2_signal intensity of the gene from all samples in order to standardize gene expression to a mean of zero. Hierarchical clustering was performed using TmeV [65] (http://www.tm4.org/) with the Euclidian distance and the complete linkage clustering parameters. KMC support parameters were used to identify clusters of genes that behave similarly in all samples. Prior identification, the optimal number of clusters was determined using the FOM (Figure of Merit) function. Differential expression patterns between conditions were identified by SAM (Significant Microarray Analysis) program. Briefly, SAM computes a statistic for each gene measuring the strength of the relationship between gene expression and the response variable. We applied a permutational test (100 permutations) to better estimate false discovery rate (FDR), which was used to estimate the proportion of false positives. Only genes with an FDR lower or equal to 5% were considered for further analyses.

#### Gene ontology annotation

Gene ontology analysis of the differentially expressed genes was performed using PANTHER. 13.1 [66, 67] and differentially expressed genes that were up and down-regulated were categorized to Biological Processes. In the case of differentially concentrated proteins, KEGG Orthology classification was used, to assign GO terms according to biological function [68]. Contingency analyses were performed on the different pathways to attest their differential representation across the different conditions (Nat. Pop, T1, T2) using JMP (version 14.1.0). Significant differences were considered when α < 0.05.

#### Degree of correlation/congruency

In order to test the degree of similarity between gene expression and protein concentration, analyses of congruency were performed in each treatment (i.e. T1 and T2). Due to the absence of replication in our proteomics analyses, correlation between proteomics and transcriptomics could not be assessed. Congruency analyses were performed as described by Mayfied and collaborator (2016) [50]. Briefly congruency was defined to occur when both proteomics and transcriptomics yielded to the same results (either +/+ or −/−). Double “non-significant” (no significant effects for proteome and transcriptome) were not considered.

## Abbreviations

DEG: Differentially Expressed Gene
G3P: Glyceraldehyde 3-Phosphate
GO: Gene Ontology
MOX: Methanotrophic symbionts
NF-KB: Nuclear Factor-Kappa B
PGRP: PeptidoGlycan Recognition Protein
PRR: Pattern Recognition Receptor
Ru5P: Ribulose-5-Phosphate
SOX: Thiotrophic symbiont
TCA: TriCarboxylic Acid
TLR: Toll-like Receptors
TNF: Tumor Necrosis Factor
